# Monitoring of Particulate Fouling Potential of Feed Water with Spectroscopic Measurements

**DOI:** 10.3390/membranes13070664

**Published:** 2023-07-12

**Authors:** Marc Weirich, Sergiy Antonyuk

**Affiliations:** Institute of Particle Process Engineering, RPTU Kaiserslautern-Landau, Gottlieb-Daimler-Straße, 67653 Kaiserslautern, Germany; sergiy.antonyuk@mv.rptu.de

**Keywords:** particulate fouling, UV–VIS spectroscopy, modified fouling index, monitoring, optical measurement

## Abstract

The modified fouling index (MFI) is a crucial characteristic for assessing the fouling potential of reverse osmosis (RO) feed water. Although the MFI is widely used, the estimation time required for filtration and data evaluation is still relatively long. In this study, the relationship between the MFI and instantaneous spectroscopic extinction measurements was investigated. Since both measurements show a linear correlation with particle concentration, it was assumed that a change in the MFI can be detected by monitoring the optical density of the feed water. To prove this assumption, a test bench for a simultaneous measurement of the MFI and optical extinction was designed. Silica monospheres with sizes of 120 nm and 400 nm and mixtures of both fractions were added to purified tap water as model foulants. MFI filtration tests were performed with a standard 0.45 µm PES membrane, and a 0.1 µm PP membrane. Extinction measurements were carried out with a newly designed flow cell inside a UV–VIS spectrometer to get online information on the particle properties of the feed water, such as the particle concentration and mean particle size. The measurement results show that the extinction ratio of different light wavelengths, which should remain constant for a particulate system, independent of the number of particles, only persisted at higher particle concentrations. Nevertheless, a good correlation between extinction and MFI for different particle concentrations with restrictions towards the ratio of particle and pore size of the test membrane was found. These findings can be used for new sensory process monitoring systems, if the deficiencies can be overcome.

## 1. Introduction

A steadily increasing demand for pure and drinking water has shifted the focus of the current research in water desalination to more energy-efficient and economic solutions [1,2]. While membrane-driven desalination processes are in the leading positions in this field, membrane fouling remains one of the challenging issues in membrane-based processes [3,4]. To assess the particulate fouling potential of the membrane systems in reverse osmosis (RO) and nanofiltration (NF) systems, the measurement of the silt density index (SDI) [5] is considered to be an industrial standard tool. Since the SDI shows poor repeatability, even with test membranes of the same batch, and is not based on any physical model [6,7], an alternative test, the modified fouling index (MFI), was proposed by Schippers et al. in 1980 [8]. The scientific community adopted the MFI after its introduction, since it has a linear correlation with the concentration of colloidal matter, temperature, and pressure compensation, and it is based on the mechanism of cake filtration. It is possible to calculate the rate of fouling for the given operating conditions of an RO plant (e.g., filtrate flux, deposition factor, temperature) [9]. The latest research suggests the utilization of MFI-UF (ultrafiltration) to assess the particulate fouling potential of RO feed waters, since this method shows a better correlation between the measured and pressure increases of real RO plants at a constant flux caused by colloidal particles than the standard MFI_0.45_ (filter medium with 0.45 µm pore size) [10,11]. Despite these advantages over the SDI, the MFI_0.45_ was only approved as a standard method by the ASTM (D8002-15) in 2015 [12]. 

Nevertheless, every current method to estimate the fouling potential requires test filtration, which leads to higher costs for a technician or an automated measurement system. In addition, the measurement result is calculated after the test filtration. In many industrial plants, only one measurement per hour, or even per day, is performed. Therefore, specific events, such as ship movements close to the inlet port of the feed pumps of salt water reverse osmosis (SWRO) plants near coasts are not represented properly in these measurements, as they were conducted in an online measurement. Experiments to correlate the SDI and online turbidity measurements with the feed water of a RO plant after pre-treatment in the Arabian Gulf by Mosset et al. [13] were not successful. The current research focuses on monitoring methods for the membrane surfaces to assess the organic or biofouling potential in membrane processes [14,15]. New research about possibilities to assess the particulate fouling potential using online monitoring techniques are rarely found. A possible technique to monitor the optical properties of a suspension is by evaluating spectroscopic (extinction) measurements with the spectral extinction method. In prior research [16], this method was used to measure the mean particle size and concentration of colloidal calcium carbonate particles during a precipitation reaction.

In this research, the capability of measuring the particulate fouling potential with the spectral extinction method was investigated. The MFI_0.45_ and MFI_0.1_ measurements were compared to the extinction of the light wavelengths of 275, 405, and 515 nm, and the extinction ratios of 275 and 515 nm. These wavelengths were chosen, since LEDs with similar light emissions are available, and the transferability of the following results towards a new sensor system with necessary adaptions is given. To take the typically low particle concentrations of RO feed water into account, adaptions to the prior setups were performed. A novel correlation between fouling potential, extinction, and extinction ratio could be shown for mono- and bimodal foulants of spherical silica particles, delivering the foundation for further research.

## 2. Models

### 2.1. Modified Fouling Index (MFI)

The MFI is commonly used to express the rate of cake formation on a membrane surface, which partially describes the flux decrease in an RO process. It is derived from filter Equation (1) and determined under the assumption that particular fouling occurs in the three stages of pore blocking, cake filtration, and cake compression [17,18].

(1) $$\frac{t}{V}=\frac{\eta \cdot R_{\mathrm{fm}}}{∆P\cdot A}+\frac{\eta \cdot \alpha \cdot C_{b}}{2\cdot ∆P\cdot A^{2}}\cdot V$$

(2) $$I=\alpha \cdot C_{b}$$

(3) $$\mathrm{MFI}=\frac{\eta \cdot I}{2\cdot ∆P\cdot A^{2}}$$

where t is time, V is the filtrate volume, η is the water viscosity, R_fm_ is the clean membrane resistance, ∆P is the transmembrane pressure, A is the membrane area, α is the specific filter cake resistance, C_b_ is a concentration constant (mass of the dried filter cake divided by the filtrate volume) in kg/m^3^, and I is the fouling index in Equation (2).

**Figure 1:**
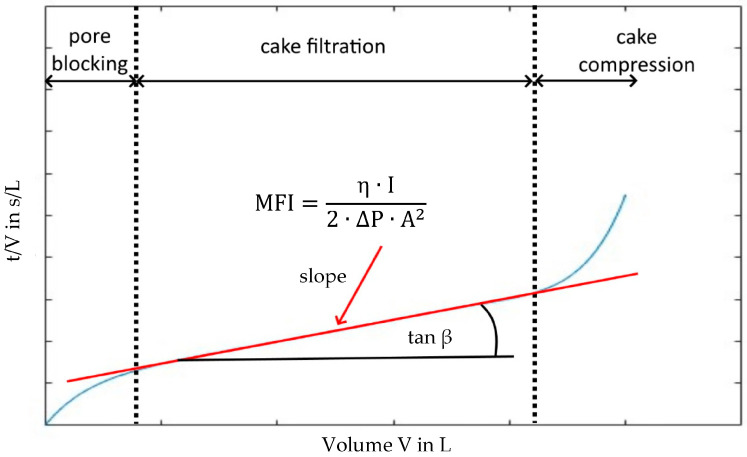
Illustration of the MFI measurement principle under constant pressure.

Equations (2) and (3) reveal the linear correlation of the MFI with both the specific filter cake resistance α and the particle concentration C_b_ derived from Equations (1) and (2). The MFI in Equation (3) is defined as the minimal slope (tan β) of the linear region of the t/V-V diagram during cake filtration over 15 min of filtration time, as shown in Figure 1 [11].

By normalizing with viscosity $\eta_{0}$, transmembrane pressure $∆P_{0}$, and membrane area $A_{0}$, the changing test conditions are compensated as shown in Equation (4):

(4) $$\mathrm{MFI}=\frac{\eta_{0}}{\eta }\cdot \frac{∆P}{∆P_{0}}\cdot {\left(\frac{A}{A_{0}}\right)}^{2}\cdot \tan\beta$$

### 2.2. Basics of the Spectral Extinction Method

#### 2.2.1. Beer–Lambert Law and Light Scattering

The extinction of a homogeneous light beam crossing a particulate system can be described by the Beer–Lambert law in Equation (5). For independent scattering, a linear relationship between the extinction of a light beam $E\left(x,c_{N},\lambda ,m\right)$, the extinction cross-section of a particle $C_{\mathrm{ext}}\left(x,\lambda ,m\right)$, and the particle concentration $c_{N}$ is provided [19,20,21].

(5) $$E\left(x,c_{N},\lambda ,m\right)=-\ln\left(T\right)=-\ln\left(\frac{I_{T}}{I_{0}}\right)=c_{N}\cdot C_{\mathrm{ext}}\cdot L_{\mathrm{mv}}$$

The extinction results from the transmission T, which is calculated by the ratio of the intensity of the transmitted light beam at the outlet of the measuring volume $I_{T}$ and the initial intensity of the light beam at the inlet of the measuring volume $I_{0}$. $L_{\mathrm{mv}}$ represents the optical path length of the light beam crossing the particulate system.

Since the Beer–Lambert law contains two unknown variables ($c_{N}$ and $C_{\mathrm{ext}}$), a second evaluation source is required to solve Equation (5). The extinction cross-section of a particle $C_{\mathrm{ext}}$ is defined in Equation (6) as the product of the extinction coefficient $k_{\mathrm{ext}}$ and the geometric particle cross-section $A_{p}$ in the beam direction, and depends on the particle size x, the wavelength of the light beam λ, and the refractive index ratio between particles and fluid m.

(6) $$C_{\mathrm{ext}}\left(x,\lambda ,m\right)=k_{\mathrm{ext}}\left(x,\lambda ,m\right)\cdot A_{p}$$

The extinction coefficient $k_{\mathrm{ext}}$, and therefore also the extinction cross-section $C_{\mathrm{ext}}$, depend on the scattering regime. The scattering regime can be determined by the size parameter $\alpha_{m}$, which is defined by the ratio of particle size x and wavelength λ of the electromagnetic wave, as follows [22]:

(7) $$\alpha_{m}=\frac{\pi \cdot x}{\lambda }$$

There are three categories for classifying the scattering regime based on the size parameter:

- $\alpha_{m}\ll 1$: Rayleigh regime
  homogeneous scattering around the particle;
- $\alpha_{m}\approx 1$: Mie regime
  complex scattering distribution around the particle;
- $\alpha_{m}\gg 1$: Fraunhofer regime
  scattering according to geometrical optics.

**Figure 2:**
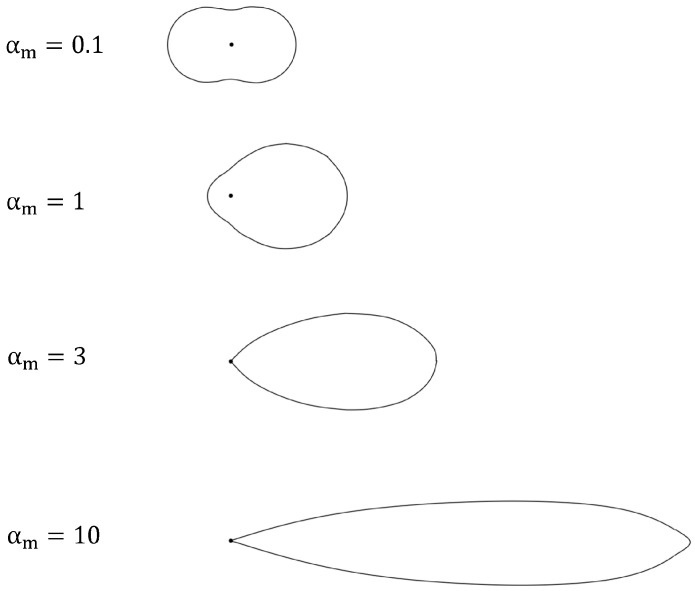
Qualitative presentation of the transition from Rayleigh (top) towards Mie scattering (bottom), with a light incident from the left of the diagram.

The different scattering principles for Rayleigh and Mie scattering are shown in Figure 2 [23]. For particle sizes around and below the wavelength of the incident light, $k_{\mathrm{ext}}$ is a function of the particle size, light wavelength, and the refractive indices of dispersed and continuous phases. For particle sizes far above the wavelength of the incident light, Fraunhofer diffraction follows the principles of classical geometrical optics. Therefore, $k_{\mathrm{ext}}$ equals one. In this research, measurements were performed with colloidal nanospheres, where $k_{\mathrm{ext}}$ can be obtained by different approaches, such as the discrete dipole approximation (DDA) or Mie’s theory [22,24]. As a result of the simplicity of the method, Mie’s theory was considered in this study. 

#### 2.2.2. Mie Theory and BH Algorithm

Mie theory describes the scattering of an electromagnetic wave at a spherical body by solving the Maxwell equations. This theoretical approach is valid for the entire electromagnetic spectrum [22]. To calculate the extinction coefficient $k_{\mathrm{ext}}$, the Bohren–Huffman (BH) algorithm is applied. It follows from the extinction or forward-scattering theorem [25,26], leading to the following:

(8) kext=2αm2∑n=1∞2n+1·Re(an+bn),

where Rez is a function returning the real part of a complex number *z*. Since all infinite series can be truncated after $n_{\max}$ terms, BH proposed for better efficiency the following limit value:

(9) nmax=αm+4·αm13+2

To obtain the Mie coefficients $a_{n}$ and $b_{n}$ from Equation (8), Equation (10) can be solved under the assumption that the magnetic permeability of particle and fluid are equal [24].

(10) $$a_{n}=\frac{m^{2}\cdot \psi_{n}\left(m\cdot \alpha_{m}\right)\cdot \left[\alpha_{m}\cdot \psi_{n}\left(\alpha_{m}\right)\right]’-\psi_{n}\left(\alpha_{m}\right)\cdot \left[m\cdot \psi_{n}\left(m\cdot \alpha_{m}\right)\right]’}{m^{2}\cdot \psi_{n}\left(m\cdot \alpha_{m}\right)\cdot \left[\alpha_{m}\cdot \xi_{n}\left(\alpha_{m}\right)\right]’-\psi_{n}\left(\alpha_{m}\right)\cdot \left[m\cdot \xi_{n}\left(m\cdot \alpha_{m}\right)\right]’}\;b_{n}=\frac{\psi_{n}\left(m\cdot \alpha_{m}\right)\cdot \left[\alpha_{m}\cdot \psi_{n}\left(\alpha_{m}\right)\right]’-\psi_{n}\left(\alpha_{m}\right)\cdot \left[m\cdot \alpha_{m}\cdot \psi_{n}\left(m\cdot \alpha_{m}\right)\right]’}{\psi_{n}\left(m\cdot \alpha_{m}\right)\cdot \left[\alpha_{m}\cdot \xi_{n}\left(\alpha_{m}\right)\right]’-\xi_{n}\left(\alpha_{m}\right)\cdot \left[m\cdot \alpha_{m}\cdot \psi_{n}\left(m\cdot \alpha_{m}\right)\right]’},$$

where prime (′) means derivative with respect to the argument. $\psi_{n}$ and $\xi_{n}$ are spherical or Riccati–Bessel functions [27]. 

#### 2.2.3. Spectral Extinction Method

In the Mie regime, for fine particles below 1–2 µm, $C_{\mathrm{ext}}$ can be derived by the ratio of at least two extinction measurements with different wavelengths of the light beam. Equation (6) links $C_{\mathrm{ext}}$ to the calculated extinction coefficient $k_{\mathrm{ext}}$ and the particle cross-section area $A_{p}$. Substituting Equation (6) into Equation (5) reveals the relationship of the measured extinction ratio of light beams $E_{i/j}$ with wavelengths i and j and the ratio of their extinction coefficients $k_{\mathrm{ext},\;i/j}$, as shown in the following equation [28,29,30]:

(11) $$\frac{E_{i}\left(x,c_{N},\lambda_{i},m\right)}{E_{j}\left(x,c_{N},\lambda_{j},m\right)}=\frac{c_{N}\cdot k_{\mathrm{ext},i}\left(x,\lambda_{i},m\right)\cdot A_{p}\cdot L_{\mathrm{mv}}}{c_{N}\cdot k_{\mathrm{ext},j}\left(x,\lambda_{j},m\right)\cdot A_{p}\cdot L_{\mathrm{mv}}}=\frac{k_{\mathrm{ext}}\left(x,\lambda_{i},m\right)}{k_{\mathrm{ext}}\left(x,\lambda_{j},m\right)}$$

**Figure 3:**
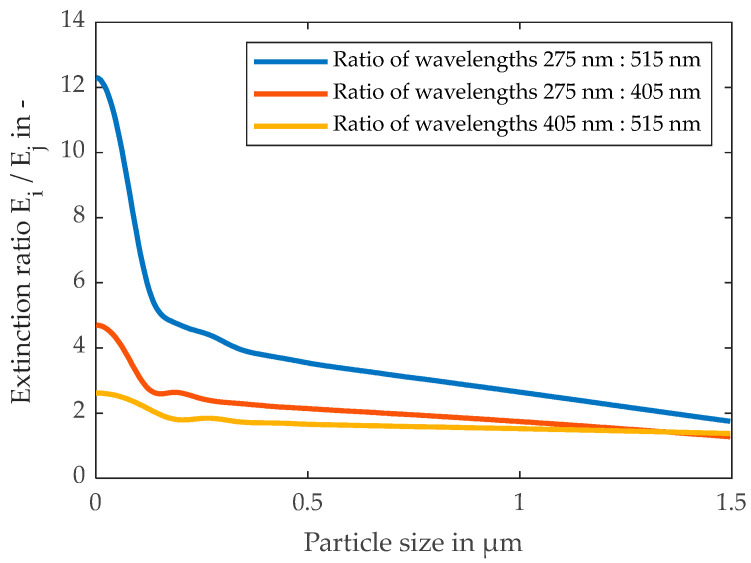
Theoretical course of the extinction ratio of monodisperse silica particles in water for wavelengths of 275, 405, and 515 nm, depending on the particle size calculated by Equations (8) and (11).

An exemplary solution for Equation (11) for a system of monodisperse, spherical silica particles and water (m = 1.43/1.33) with neglected absorption is shown in Figure 3. It is possible that no explicit particle size can be calculated by one extinction ratio. Therefore, the utilization of further extinction ratios of more wavelengths can eliminate this ambiguity, as explained in [16]. In the validity range of the Beer–Lambert law, an explicit mean particle size and mean particle concentration can be determined [28,29]. 

### 2.3. Modified Stöber Process

Monodisperse, spherical silica colloids are produced with the Stöber process by means of the hydrolysis of alkyl silicates and polycondensation of silicic acid in alcoholic solutions, usually ethanol and water, along with an ammonia catalyst. The diameter of silica particles is controlled by the relative contributions of nucleation and growth rate, which depend on the reaction temperature and the concentration of the reactants [31].

During the process, a hydrolysis reaction described in Equation (12) is followed by a condensation reaction, as shown in Equation (13). During the hydrolysis, tetraethylorthosilicate (TEOS) reacts with water into mono-silicic acid and ethanol. In this modified Stöber process the additional catalyst tetramethylenediamine (TMED) is added along with ammonia. The catalysts increase the number of hydroxide ions, which leads to an increased hydrolysation rate [32,33]. The alcoholic solution additionally serves as a solvent for the insoluble in water TEOS.

(12) $$\mathrm{Si}{\left({\mathrm{OC}}_{2}H_{5}\right)}_{4}+4H_{2}O\overset{\;\;\;\;\;\;\;\;\;\;\;\;\;\;\;}{\to }\mathrm{Si}{\left(\mathrm{OH}\right)}_{4}+4C_{2}H_{5}\mathrm{OH}$$

(13) $$2\mathrm{Si}{\left(\mathrm{OH}\right)}_{4}\underset{-H_{2}O}{\to }{\left(\mathrm{OH}\right)}_{3}\mathrm{Si}–O–\mathrm{Si}{\left(\mathrm{OH}\right)}_{3}\overset{Polycondensation}{\underset{-H_{2}O}{\to }}{\mathrm{SiO}}_{2}$$

Following the hydrolysis, a condensation reaction occurs, as water molecules are removed from the mono-silicic acid while disilicic acid is formed. In the next polycondensation step, more water molecules are removed from the remaining silicic acid and silica particles are formed. Since the rate of condensation is much higher than the rate of hydrolyzation, hydrolyzation is identified as the reaction-determining step of Stöber’s process [32]. Both reactions end, once the reacting ingredients are consumed [34].

## 3. Materials and Methods

### 3.1. Chemicals for the Particle Synthesis by the Modified Stöber Process

The following materials (Table 1) were used for the synthesis of the silica nanoparticles. 

**Figure 4:**
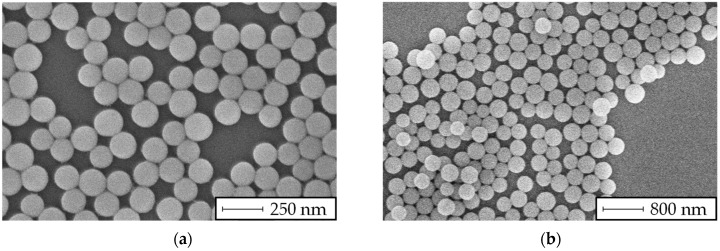
SEM images of (**a**) 120 nm and (**b**) 400 nm silica particles.

The reaction conditions shown in Table 2 were set to produce two fractions of particles. The reactants were filled into a 250 mL reaction flask, which was indirectly heated by a water bath on a magnetic stirrer hot plate. After the reaction time elapsed, the solvents were drawn from the suspension by a rotary evaporator (Carl Roth, Karlsruhe, Germany, RV 3 V). Further DI-water was added to adjust the particle concentration to about 10 wt%. The crafted particle suspensions were measured with a static light scattering (SLS) sensor (Malvern, Kassel, Germany, Horiba LA-950), resulting in mean particle sizes of 120 nm with a standard deviation of ±10 nm, and 400 nm with a standard deviation of ±80 nm, as shown in Figure 4.

### 3.2. Materials for MFI Testing

The following materials were used to conduct the MFI filtration tests: a polyethersulfone (PES) membrane with a nominal pore size of 0.45 µm (3M Membrana, Wuppertal, Germany, DuraPES 450) and a poly-propylene (PP) membrane with a nominal pore size of 0.1 µm (3M Membrana, Germany, Accurel PP-01). Tap water was post-treated with a dialyzer with a nominal pore size of 5.5 nm (Inuvai, Bad Homburg, Germany, R180) to ensure that the water for the experiments was free of particles. The PES membranes were soaked in DI water for at least 30 min before filtration to minimize swelling effects. The hydrophobic PP membranes were wetted with ethanol prior to the experiments to improve their flowability with water.

### 3.3. Experimental Setup for Combined Fouling Potential Measurements and Test Conditions

**Figure 5:**
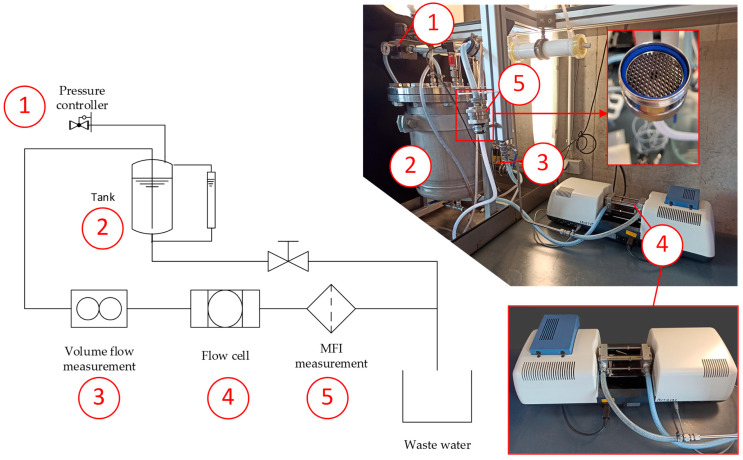
Experimental setup for the simultaneous measurement of fouling potential and optical extinction.

To combine the MFI and spectral extinction measurement, a dead-end filtration test bench was set up, according to Figure 5. The stainless steel filter holder (pos. 5) carried membrane samples with a 47 mm diameter. The silica particles were dispersed in filtered water inside a 27 L stainless steel tank (pos. 2). The feed water tank was pressurized (pos. 1) with filtered air at 2.07 bar (30 psi) to supply the transmembrane pressure for constant pressure filtration. Since colloidal particles have a low sedimentation velocity of a few mm per minute, a mixing or stirring system inside the tank was renounced.

**Figure 6:**
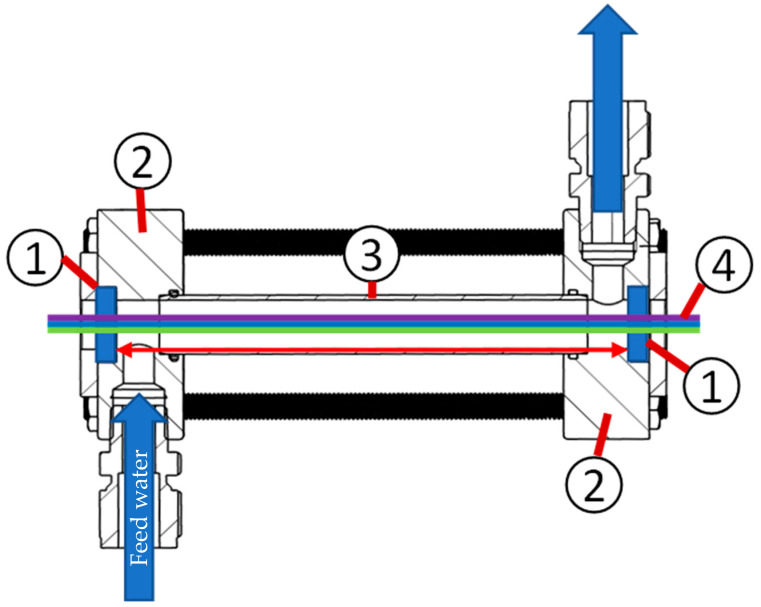
Section view of the flow cell, light path, and flow direction of the model foulant (feed).

The filtrate flow was measured using a flow meter (pos. 3) (Bürkert, Ingelfingen, Germany, SE-55). The optical properties of the feed water were monitored with a flow cell (pos. 4), as shown in Figure 6, to obtain optical access for the UV–VIS spectrometer (Analytic Jena, Jena, Germany, Specord S600). The developed flow cell provided an adjustable optical path length in the range of 40 mm to 150 mm. The maximum length was limited by the UV–VIS spectrometer. The windows (pos. 1), which grant optical access for the light rays (pos. 4) to screen the feed water, were made of quartz glass, which ensures a high transmission in the deep ultraviolet (UV-C) and VIS spectra. The windows were fitted inside two connecting plates (pos. 2) for inserting pipes (pos. 3) of different lengths to adjust the optical path length. For foulants or particle systems with higher concentrations, shorter optical path lengths were advised to ensure independent scattering with higher light transmission levels to stay in the validity range of the Beer–Lambert law. If low light transmission levels are necessary, the validity range of the Beer–Lambert law can be extended with a spatial filter to eliminate multi-scattered light rays [35]. The measurement data were recorded using a data acquisition system (Beckhoff, Verl, Germany, CX5130, and Twincat 3).

Experiments at various particle concentrations were performed with monomodal silica particles of particle sizes of 120 nm and 400 nm, as well as bimodal mixtures of those particles (See Supplementary Materials). Two types of membranes with pore sizes of 0.45 µm and 0.1 µm, referred to as MFI_0.45_ and MFI_0.1_ were used. The filtration time was set to 15 min according to the standard in [12]. The test parameters are summarized in Table 3.

## 4. Results

In this chapter, the results of experiments with mono- and bimodal silica foulants are presented. The general test conditions were specified in the previous chapter.

### 4.1. Experiments with Monomodal Silica Foulants

#### 4.1.1. Light Transmission of the Model Foulants

**Figure 7:**
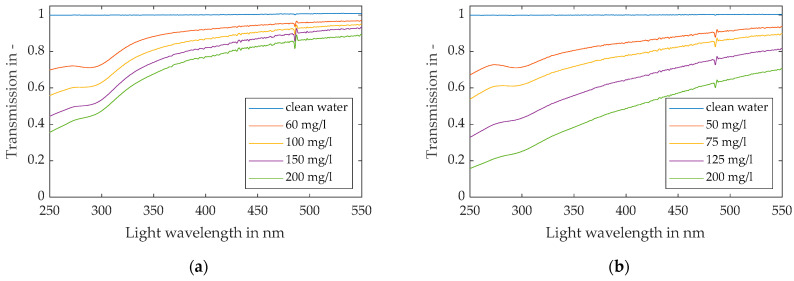
Light transmission over light wavelength for (**a**) 120 nm particle suspensions and (**b**) 400 nm particle suspensions at various particle concentrations.

The measured transmission profiles of monomodal spherical silica foulants in relation to clean water at different particle concentrations are shown in Figure 7. The concentrations ranged from 60 mg/L to 200 mg/L for the 120 nm silica particles (a), and from 50 mg/L to 200 mg/L for the 400 nm particles (b). The profiles represent the mean values of 15 measurements with a one-minute time interval. The standard deviation for the transmission was below 1% in all of the experiments, which indicates that the suspension was stable, and no agglomeration or sedimentation inside the feed tank occurred.

The obtained results indicate that different particles are distinguishable by the course of their transmission profile, as the smaller 120 nm particles show lower transmission levels in the UV light spectrum (250–400 nm) in relation to the VIS light spectrum (>400 nm) than the larger 400 nm particles. This behaviour is shown by the steeper slope of the graphs in the transition from UV to VIS range in plot (a) compared to plot (b) of Figure 7. Furthermore, an increasing particle concentration is detectable by the reduction in transmission at a specific wavelength of light, as stated by the Beer–Lambert law.

#### 4.1.2. MFI Measurement with 0.45 µm PES Membrane

Simultaneous with the optical measurement in the flow cell, the MFI was determined for various model foulants by test filtrations. The concentrations of silica particles with particle diameters of 120 and 400 nm used in the experiments are shown in Table 4.

**Figure 8:**
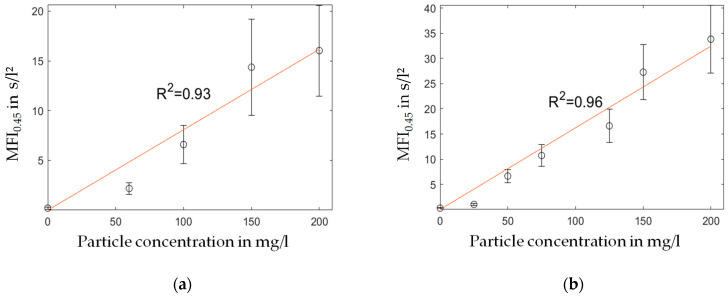
MFI over particle concentration for foulants with a particle size of (**a**) 120 nm and (**b**) 400 nm, and linear regression of the measured data.

Figure 8 shows a good linear correlation between the silica particle concentration and the observed MFI_0.45_, which is in agreement with Equations (1)–(3). Three sets of measurements were performed for every particle concentration.

#### 4.1.3. Comparison of MFI_0.45_ and Spectroscopic Measurements

**Figure 9:**
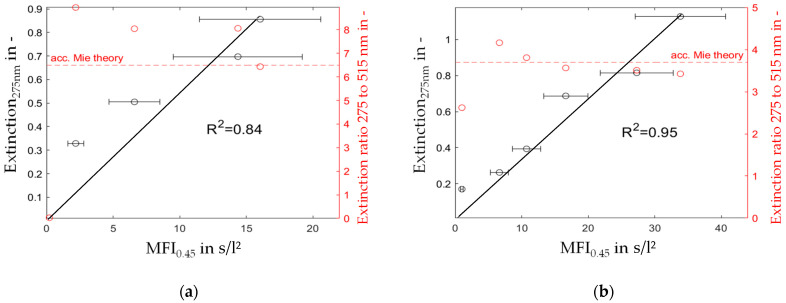
Extinction of 275 nm light (left axis) and extinction ratio of 275 and 515 nm (right axis) over MFI for foulants of a particle size of (**a**) 120 nm and (**b**) 400 nm.

The combination of spectroscopic and fouling potential (MFI) measurements in a single diagram for the particle sizes and concentrations specified in Table 4 is presented in Figure 9. By plotting the extinction of the 275 nm UV light wavelength on the left y-axis, and the extinction ratio of 275 to 515 nm light wavelengths and the calculated extinction ratio according to the Mie theory on the right y-axis, while the MFI is drawn on the x-axis as shown in Figure 3, a good correlation was observed between the MFI and UV_275nm_ extinction with a coefficient of determination R^2^ of 0.84 for the 120 nm silica particles and R^2^ of 0.95 for the 400 nm foulant.

Moreover, the extinction ratio remains consistent despite increasing MFI levels and varying particle concentrations. Deviations, mainly for the 120 nm particles are explained by the steep course of the extinction ratio over particle size in the region below 150 nm presented in Figure 3, where even small measurement deviations cause a significant error in the calculation of the particle size. Furthermore, when dealing with lower particle concentrations, the transmission of visible light still remains above 90%. This can result in notable inaccuracies in measurements [35,36].

**Figure 10:**
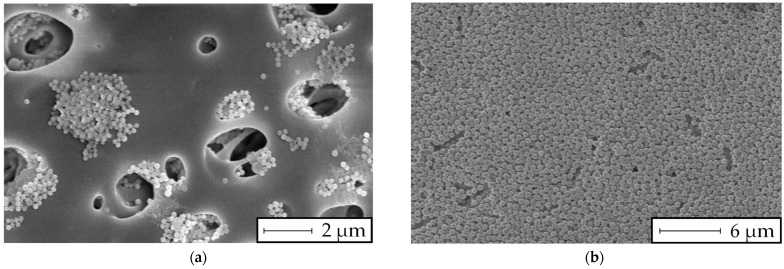
SEM images of the test membrane after 15 min of filtration for (**a**) 120 nm particles at 150 mg/L and (**b**) 400 nm particles at 50 mg/L.

After the filtration tests, SEM images of the filter cake formation of two experiments with particle sizes of 120 nm and 400 nm were made, as shown in Figure 10.

It was revealed that the particles larger than the filter medium’s pore size build a filter cake on the surface of the membrane, while the smaller particles seem to block the pores inside the membrane, resulting in depth filtration.

### 4.2. Experiments with Bimodal Silica Foulants

#### 4.2.1. Comparison of MFI_0.45_ and Spectroscopic Measurements

As stated in the previous chapter, a foulant consisting of monomodal spheres shows a good correlation between the MFI and light extinction. In this section, bimodal foulants are investigated. While the filter cake resistance was predominantly affected by smaller particles that can block the pores of the filter cake and increase the filtration resistance and consequently the MFI, larger particles had a stronger impact on the shape of the light transmission profile during the UV–VIS measurement. Therefore, the monodispersed spherical silica particles from the last chapter were mixed in the weight ratios of 3:1, 1:1, and 1:3 as a bimodal model foulant in various concentrations.

**Figure 11:**
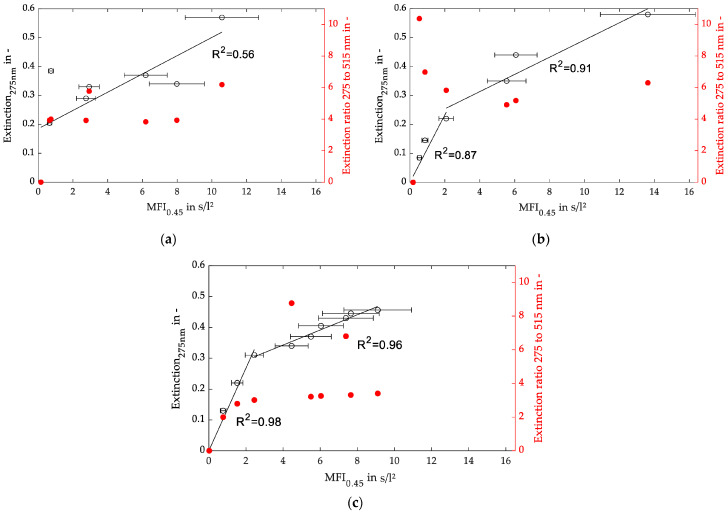
Extinction of 275 nm light (left axis) and extinction ratio of 275 and 515 nm (right axis) over MFI_0.45_ for bimodal foulants with weight ratios of 120 and 400 nm particles of (**a**) 3:1, (**b**) 1:1, and (**c**) 1:3.

While the monomodal foulant showed a strictly linear correlation between MFI_0.45_ and extinction, the results for the bimodal foulants presented in Figure 11 imply a particle size distribution and concentration dependency of the filter cake formation. The linear correlation over the particle concentration field shown in Figure 9 shifts towards a non-linear correlation, with two regions of different pitches.

Diagrams (b) and (c) of Figure 11 with a more balanced particle distribution can be divided into two regions with a good linear fit. Contrary to that observation, chart (a), with the lowest relative number of large particles, shows a moderate linear correlation between the MFI_0.45_ and UV_275nm_ extinction with an R^2^ value of 0.56, similar to the results of Figure 9a.

**Figure 12:**
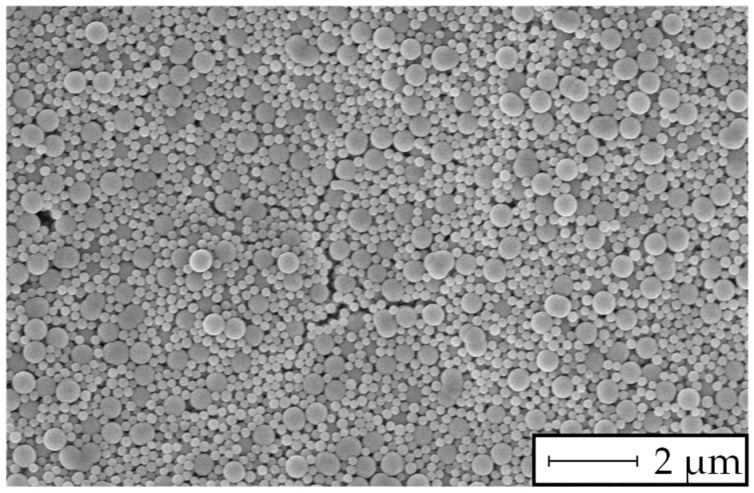
SEM image of the 0.45 µm test membrane after 15 min of filtration for a mixture of 120 nm and 400 nm particles (mass ratio 1:1) at 75 mg/L.

A SEM image of the filter cake after 15 min of filtration for a mixture of particles with a mass ratio of 1:1 is shown in Figure 12. A significant retention of particles smaller than the filter medium’s pore size was found.

Observing the extinction ratio of 275 nm and 515 nm light wavelengths, a rather constant course with some outliers can be assumed. Nevertheless, a shift in the particle size distribution towards larger particle sizes should lead to a lower extinction ratio. This behavior could not be shown in the experiments. A closer look into the transmission profiles showed that the extinction values of the 515 nm light were rather low and unstable. Therefore, the calculation of the extinction ratios may be afflicted with a large error of unknown quantity.

#### 4.2.2. Comparison of MFI_0.1_ and Spectroscopic Measurements

To investigate the influence of the filter media pore size on the filtration process, further MFI_0.1_ tests were performed with the previously used bimodal suspensions. The consideration was to explore the impact of particles smaller than the filter media’s pore size on the linkage between fouling potential and optical measurements.

**Figure 13:**
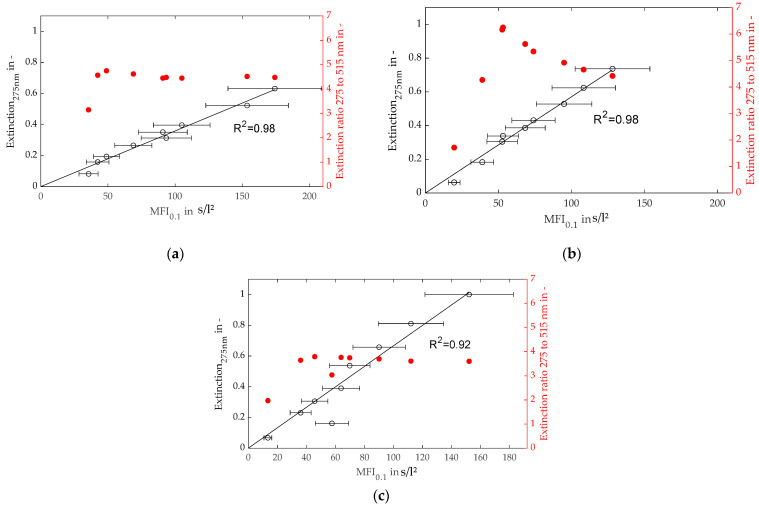
Extinction of 275 nm light (left axis) and extinction ratio of 275 and 515 nm (right axis) over MFI_0.1_ for bimodal foulants with weight ratios of 120 and 400 nm particles of (**a**) 3:1, (**b**) 1:1, and (**c**) 1:3.

Contrary to the experiments shown in Section 4.2.1 a strong linear behavior did occur, and a good correlation between the MFI_0.1_ and UV extinction for each particle concentration and ratio of the mixture could be shown, as illustrated in Figure 13. 

## 5. Discussion

The results in Figure 8 show a linear increase in the MFI over rising particle concentration levels. Consequently, the extinction measurements and the MFI_0.45_ have a similar relation, as stated by the Beer–Lambert law (Figure 9). However, for the filtration experiments with particles smaller than the filter pore size (Figure 8a and Figure 9a), a significant offset towards the intercept of the x-axis was observed. A possible explanation is the insufficient filter cake build-up during the 15 min filtration period demanded by the MFI test guidelines according to the ASTM. Otherwise, for particles with a size similar to or larger than the filter media’s pore size, the course of the diagram in Figure 9b showed a strictly linear correlation, even at low particle concentrations. In our previous study [37], the formation and collapse of particle bridges above filter medium pores during surface filtration was described with a simulation model. The simulation showed that even particles that were significantly smaller than the pores of the filter media formed bridges and consequently a filter cake, due to the adhesive forces between particle–particle and particle–filter media. The rupture and collapse of newly formed bridges is a possible mechanism to explain the inconsistent filter cake buildup in the first stage of the cake filtration process, with inconsistent throughput of particles through the filter media. This assumption may be supported by the SEM pictures presented in Figure 10 and Figure 12, as different mechanisms of filter cake buildup are implied.

In regard to the theory for cake filtration [17,38,39], only a linear correlation between particle concentration of the feed and filter cake resistance was found if the number of particles passing the filter medium was neglectable and the filter cake behaved as incompressible. Consequently, higher particle concentrations may lead to a sufficient filter cake buildup with a high retention of particles, hence complying with the filtration theory. Similar findings were reported by [40] where multiple MFI measurements with membranes of pore sizes up to 100 kDa were performed to ensure a better retention of the finest colloids, which resulted in a better correlation between the MFI and the fouling rate of an RO system. The results presented in Figure 11 with a bimodal foulant support the assumption that small particles at low concentrations interfere strongly with the linearity of the MFI_0.45_ standard described in the ASTM as the first region of diagrams (a) and (b) has a different slope than the second region. A possible explanation might be different filtration mechanisms on the microscale. At low particle concentrations, pore blocking of large particles has a huge contribution to the filter cake resistance, while small particles pass through the pores. At higher particle concentrations, the 15 min filtration time is sufficient for the formation of a cake layer with the ability to withhold even the smaller particles, leading to an increase in filter cake and specific filter cake resistance during the test.

Since the MFI is a measurement to evaluate the fouling potential in RO membranes, it can be assumed that the occurrence of particles smaller than the pore size of the test membrane at low particle concentrations causes a significant error in the assessment of the fouling rate. According to the fouling model of Schippers et al. [9], it is advised to utilize an ultrafiltration-based MFI (MFI-UF) to assess the fouling potential of practical feed waters in RO applications. The results presented in Figure 13 support this recommendation, with the presented strictly linear correlation of particle concentration and MFI_0.1_ for particles larger than the pore size of the test filter. Furthermore, the presented optical measurements are capable of monitoring changes in the fouling potential of feed waters. In future research, that finding may lead to longer times between single MFI measurements in the field and continuous monitoring of the RO feed, since fouling potential measurements in a fixed interval, e.g., once per hour or day, would lose their necessity.

The extinction ratios of two different light wavelengths for the three bimodal foulants are shown in Figure 13. According to the theory of the spectral extinction method, a mean particle size is calculatable by those values. For the plots of Figure 13b,c, the extinction ratio decreases from about 4.5 to 3.7 with an increasing fraction of large particles. Therefore, the changing filter cake composition and fouling potential are already detectable in the feed. However, a significantly larger fraction of small particles, as shown in plot (a), only caused an increase in the extinction ratio to about 4.7. Consequently, the suspensions from plots (a) and (b) are hardly distinguishable. This phenomenon may be caused by the greater shading effect of the larger particles, and their contribution to the overall extinction.

The root of those non-conform measurements in regard to the presented theory may also be justified by the UV–VIS sensor setup that caused high transmission levels, in particular with visible light, resulting in a non-neglectable measurement error. To improve the measurement capabilities of the spectral extinction method, and therefore the ability to estimate the specific filter cake resistance, further studies with a different sensor setup with increased optical path lengths are required.

## 6. Conclusions

Membrane fouling leads to higher energy consumption and less efficiency of RO processes. In this study, a method for online monitoring of the particulate fouling potential, and consequently a possibility to estimate the future fouling rate, was presented. The following conclusions could be drawn from the experimental findings:

- Online monitoring of the particulate fouling potential of RO feedwater is theoretically possible as shown through measurements with a silica model foulant.
- Different foulants and therefore specific filter cake resistances are distinguishable by the extinction ratio of different wavelengths. Yet, the results show a significant measurement error and the test setup needs improvement.
- A linear correlation between MFI and the extinction of UV_275nm_ light is given as long as sufficient filter cake is formed during the test filtration.
- The shown correlation improves, if the test membrane’s pore size is smaller than the smallest particle fraction of the feedwater. Therefore, it is advisable to perform an MFI-UF test instead of the standardized MFI_0.45_ test to evaluate the fouling potential of real-world RO feed water.

In future investigations, an adapted flow cell or sensor setup is recommended to guarantee more stable measurements and shift the confidence range towards lower particle concentrations, which are common in nanofiltration and reverse osmosis applications.

## Figures and Tables

**Table 1:** Chemical reactants and their function inside the Stöber process.

Chemical	Ammonia	Ethanol	TEOS	TMED	Isopropanol	Di water
**Purity**	25 wt%	99.9%	>99%	>99%	99.5%	< 0.1 µS/cm
**Supplier**	Grüssing GmbH, Filsum, Germany	Th. Geyer GmbH & Co. KG, Renningen, Germany	Sigma-Aldrich Chemie GmbH, Taufkirchen, Germany	Merck Schuchardt OHG, Hohenbrunn, Germany	Sigma-Aldrich Chemie GmbH, Germany	RO system + mixed bed desalination
**Function**	Catalyst	Co-solvent	Silicon source	Catalyst	Co-solvent	Solvent

**Table 2:** Reaction parameters for the Stöber process.

Particle Size	120 (±10) nm	400 (±80) nm
Reaction temperature	55 °C	30 °C
Reaction time	1 h	2 h
Stirrer speed	900 rpm	900 rpm
Ethanol	-	70 mL
Isopropanol	70 mL	-
DI-water	25 mL	10 mL
Ammonia solution	1.5 mL	30 mL
TEOS	6 mL	12 mL
TMED	0.4 mL	3 mL

**Table 3:** Test parameters for the MFI filtration and the optical measurement.

MFI Test Parameters			Optical Parameters		
Test duration:		15 min	Measurement interval:		60 s
Filter media pore size:		0.45 µm or 0.1 µm	Optical path length:		150 mm
Filter media diameter:		47 mm	Light wavelengths:		275, 405 and 515 nm
Filtration pressure:		2.07 bar (30 psi)			
Feed temperature:		21 °C			

**Table 4:** Particle concentrations of the modal foulants.

Mean Particle Size in nm:		120 (±10)	400 (±80)
		60	25
		100	50
Particle concentration in mg/L:		150	75
		200	125
			150
			200

## Data Availability

Not applicable.

## Supplementary Materials

The following supporting information can be downloaded at: https://www.mdpi.com/article/10.3390/membranes13070664/s1, Figures S1–S3: Filtration curves of 120 nm silica particles; Figures S4–S7: Filtration curves of 400 nm silica particles; Figures S8–S16: Filtration curves of mixtures of 120 nm and 400 silica particles.
